# Choroidal thickness in school children: The Gobi Desert Children Eye Study

**DOI:** 10.1371/journal.pone.0179579

**Published:** 2017-06-15

**Authors:** Dan Zhu, Yan Wang, Yan Fei Zheng, Da Yong Yang, Kai Guo, Xian Rong Yang, Xin Xia Jing, Ian Y. Wong, Qi Sheng You, Yong Tao, Jost B. Jonas

**Affiliations:** 1 The Affiliated Hospital of Inner Mongolia Medical University, Hohhot, Inner Mongolia, China; 2 Department of Ophthalmology, The University of Hong Kong, Hong Kong, China; 3 Beijing Institute of Ophthalmology, Beijing Tongren Hospital, Capital Medical University, Beijing, China; 4 Jacobs Retina Center, Shiley Eye Center, University of California San Diego, United States; 5 Department of Ophthalmology, Beijing Chaoyang Hospital, Capital Medical University, Beijing, China; 6 Department of Ophthalmology, Medical Faculty Mannheim of the Ruprecht-Karls-University Heidelberg, Mannheim, Germany; Wenzhou Medical University, CHINA

## Abstract

**Purpose:**

To investigate choroidal thickness (CT) and its associations in children in a school-based study.

**Methods:**

The cross-sectional school-based Gobi Desert Children Eye Study included 1565 out of 1911 (81.9%) eligible children from all schools in the oasis region of Ejina in the Gobi Desert. A detailed ophthalmic examination was performed, including spectral-domain optical coherence tomography with enhanced depth imaging for CT measurement.

**Results:**

CT measurements were available for 1463 (93.5%) students (mean age: 11.8±3.5 years; range:7–21 years). Mean subfoveal choroidal thickness (SFCT) was 282±49μm. CT was thickest at 1000μm temporal to the fovea (286±49μm), followed by the subfoveal region (282±49 μm; *P*<0.001), the region at 2500μm temporal to the fovea (278±49μm), the region at 1000μm nasal to the fovea (254±49μm;*P*<0.001), and the region at 2500μm nasal to the fovea (197±50μm;*P*<0.001). In cross-sectional analysis, the mean SFCT increased with age from 288μm at 7 years of age to 304μm at 11 years, and then decreased to 258 μm at 18 years. In multivariate analysis, thicker SFCT was associated (regression coefficient r:0.38) with higher hyperopic refractive error (*P*<0.001;standardized regression coefficient beta:0.31;non-standardized regression coefficient B:7.61;95% confidence intervals (CI):6.29,8.93), younger age (*P*<0.001;beta:-0.10;B:-1.39;95%CI:-2.14,-0.64), male gender (*P* = 0.03;beta:-0.05;B:-5.33;95%CI:-10.1,-0.53), higher corneal refractive power (*P*<0.001;beta:0.12;B:3.68;95%CI:2.12,5.24), and non-Han Chinese ethnicity (*P* = 0.03;beta:0.05;B:6.16;95%CI:0.50,11.8). Ratio of CT(1000μm nasal to fovea)/SFCT (0.90±0.06;range:0.66,1.23) and ratio of CT(2500μm nasal to fovea)/SFCT (0.70±0.13;range:0.28,1.23) decreased with older age (*P* = 0.01;and *P* = 0.001, respectively), while ratio of CT(1000μm temporal to fovea)/SFCT (1.02±0.06;range:0.56,1.37) and ratio of CT(2500μm temporal to fovea)/SFCT (0.99±0.11;range:0.54,1.84) increased with older age (both *P*<0.001). Time spent outdoors or indoors was not significantly associated with CT-related parameter in multivariate analysis.

**Conclusions:**

In contrast to SFCT in adults and despite elongating axial length, SFCT in children increased in cross-sectional analysis with older age (up to 11 years of age) and then started to decrease with further ageing. It suggests an increase in choroidal volume up to the age of 11 years. In children, the choroid was thickest at 1000μm temporal to the fovea, followed by the subfoveal region, and this difference significantly increased with older age. In contrast, CT nasal to the fovea in relationship to SFCT decreased with older age. CT was independent of lifestyle-associated parameters.

## Introduction

The choroid is comprised of blood vessels, melanocytes, fibroblasts, resident immunocompetent cells and supporting collagenous and elastic connective tissue. The clinical significance of the choroid includes nourishing the outer retinal layers, regulating temperature and involvement in the pathogenesis of many blinding diseases such as age-related macular degeneration, polypoidal choroidal vasculopathy, Vogt-Koyanagi-Harada disease and myopic retinopathy [1]. Studies also suggested that the subfoveal choroid may intricately be involved in the development of myopia, one of the most common eye disorders worldwide nowadays [2,3,4].

Choroidal thickness can be measured noninvasively *in vivo* using spectral-domain optical coherence tomography (OCT) [5]. Using enhanced depth imaging technique, previous studies on adults have reported the normal values of subfoveal choroidal thickness (SFCT) and their associations with age, refractive error, axial length and gender [6–11]. Clinical studies on adults showed that lower best corrected visual acuity was associated with a thinner choroid (also called leptochoroid), in particular with a subfoveal choroid thinner than 30 μm [12], and that some macular diseases are associated with an abnormal SFCT. To cite examples, patients with central serous chorioretinopathy had a thickened SFCT in the affected eye as well as in the contralateral unaffected eye [13,14], patients with polypoidal vascular choroidopathy showed an increased SFCT in association with a dilatation of the large choroidal vessels [15–18], patients idiopathic subfoveal choroidal neovascularization showed an abnormally thick SFCT which decreases after intravitreal ranibizumab injection [19,20], patients after a non-arteritic anterior ischemic optic neuropathy had an abnormally thin SFCT [21], and patients with age-related macular degeneration, long-standing retinal vein occlusions, open-angle glaucoma or with diabetes and diabetic retinopathy exhibited a normal thickness of the subfoveal choroid [22–25]. In pilot studies, SFCT was additionally correlated with estimated cerebrospinal fluid pressure and cognitive function in adults [26,27]. Choroidal thickness in the subfoveal region and in the peripapillary region decreased significantly parallel to an acute increase in intraocular pressure in a dark room adaptation test [28]. Some recent investigations on children suggested that the morphology of the choroid and its associations with ocular and general parameters differed between adults and children [29–31].

Despite the large volume of studies, including population-based investigations, on choroidal thickness in adults, there is a paucity of data on choroidal thickness in children recruited in population-based or school-based studies. Most of the previous studies on children were hospital-based and had a relatively small sample size, leading to a potential bias by a referral bias [29–37]. We therefore conducted the present study to examine the choroidal thickness in children with recruitment of the study participants on the basis of school attendance and to assess associations of choroidal thickness with other ocular and general parameters. We choose as study site an oasis city in the Gobi Desert, with the next settlement located at a distance of about 400 km. The advantage of this study site was that all schools in the oasis city could be included into the study and that due to the low mobility of the study participants and their families to move out of the city, the city population had remained constant for a relatively long time. Since the population of the city included Mongolian descendants as well as Han Chinese, the study design additionally offered the possibility of an inter-ethnic comparison. Since choroidal thickness is markedly affected by refractive error, since previous studies suggested a potential role of the choroid in the process of myopization, and since recent investigations showed an association between school children myopia and lifestyle, we also examined lifestyle-related parameters such as the ratio of indoors to outdoors activities and the level and intensity of education, to test the hypothesis whether staying predominantly indoors versus outdoors or whether the level of education was associated choroidal thickness [2,7–12,38].

## Methods

The Gobi Desert Children Eye Study was a school-based cross-sectional study performed in the city oasis of Ejina, locating in the most western part of the Chinese province of Inner Mongolia at 100.90° to 101.42° East longitude and from 41.85° to 42.50° North latitude. With extremely arid conditions, the study area belongs to the north temperature climate zone with a mean annual precipitation of approximately 40 mm and a mean pan evaporation of 3700 and 4000 mm. Average winter temperature minimums are close to −40°C, while summertime temperatures are warm to hot, with highs that range up to 50°C. The next settlement is located in a distance of approximately 400 km. Ejina can be reached by train (15 hours from Hohhot, the capital of Inner Mongolia) and by road. The study complied with the Helsinki declaration and was approved by the Ethics Board of the Affiliated Hospital of Inner Mongolia Medical University Hohhot and the local Administration of the Education and School Board of Ejina. Written informed consent was obtained from the parents or guardians of all children.

The study has been described in detail previously [39,40]. In short, the study included all three available schools in Ejina, the Ejina Primary School (911 students), the Ejina Middle school (765 students), and the Ejina Minority School (235 students)), and consisted of altogether 1911 children. There was no exclusion criterion. The ophthalmological examinations included assessment of best corrected visual acuity, slit lamp-based examination of the anterior ocular segment by an ophthalmologist, tonometry (non-contact tonometer; Canon TX-F Full-Auto Tonometer, Canon Co., Tokyo, Japan), and examination of ocular motility, binocularity and presence of strabismus. Cycloplegic refractometry was performed using an auto-refractometer ion (ARK-900, NIDEK, Tokyo, Japan) after instilling 1% cyclopentolate eye drops (Alcon, Ft. Worth, USA) at least three times. Refractometry and tonometry were performed three times and the mean value of the measurements was recorded and used for statistical analysis. Fundus photography and optical coherence tomography were carried out after pupil dilation. Systemic examinations included measurement of body height (using a stadiometer) and body weight, heart rate and blood pressure (using an automatic blood pressure monitor (YE655A, YUYUE, Jiangsu, China)). The parents of the children were interviewed using a standardized questionnaire which included questions on the profession, level of education, income and ethnic background of both parents, the birth weight, birth age and type of birth of the children, and whether oxygen was supplied after birth. Glasses worn by the parents were measured as an estimate of their refractive errors. The interview also included questions on the number of school days per week, sleeping time and duration, time spent outdoors before going to school, time, duration and type of travelling to and from school, time spent outdoors at school before the school started, time and duration of school work, time spent outdoors at school and time spent outdoors at home after finishing the school work, type of activities when being outdoors, and time spent indoors with reading or writing for school work, reading at pleasure, working on the computer, watching television, indoors sport or other indoors activities.

Spectral domain optical coherence tomography (Spectralis^®^, Wavelength: 870nm; Heidelberg Engineering Co., Heidelberg, Germany) with enhanced depth imaging modality was performed after pupil dilation. The horizontal section running through the center of the fovea was selected for measurement of choroidal thickness, which was defined as the vertical distance between the hyperreflective line of Bruch’s membrane and the hyperreflective line of the inner surface of the sclera. The measurements were carried out using the built-in software. For each eye, choroidal thickness was measured at five locations: subfoveal, at 1000μm and at 2500μm nasal to the fovea, and at 1000μm and at 2500μm temporal to the fovea.

The spherical equivalent of the refractive error was defined as the spherical value of refractive error plus one half of the cylindrical value. Body mass index was calculated as the ratio of body weight (expressed in kg) divided by the square of body height (expressed in meter). The mean arterial blood pressure was defined as diastolic blood pressure plus one third of the difference between systolic blood pressure and diastolic blood pressure. Myopia was defined as a spherical equivalent of refractive error of ≤-0.50 diopters.

Statistical analysis was carried out using the SPSS-for-Windows software (version 22.0; IBM-SPSS, Chicago, IL, USA). Descriptive statistics included mean, standard deviation, median, range, and percentages and were presented where appropriate. The normal distribution of parameters was tested by the Kolmogorov-Smirnov test. In the case of not normally distributed parameters, the Mann-Whitney test was applied to examine the statistical significance of differences between un-paired groups. The Chi-square test was used to compare proportions. The paired Student´s-t-test was applied to compare choroidal thickness measured at different locations of the same eye or to assess the inter-eye difference of the same individual. Linear regression analysis was applied to examine associations between choroidal thickness and other parameters such as age, refractive error and body mass index. *P*-values represented results for 2-sided tests, with values less than 0.05 considered statistically significant.

## Results

Out of 1911 primarily eligible children, 346 refused the examination, so that 1565 (81.9%) children eventually participated in the study, among whom 1463 (93.5%) children underwent EDI OCT examination for measuring choroidal thickness. The mean age of the 1463 participants [746 (51.0%) boys] was 11.8 ± 3.5 years (median: 11.5 years; range: 7 to 21 years). There were 1127 (77.0%) Han students and 336 (23.0%) students of non-Han Chinese ethnicity including Mongolian, Hui, Man, Tibetan and Tujia. Mean refractive error (spherical equivalent) was -1.20 ± 2.03D (median: -0.63 diopter, range: -12.75 to +6.63 diopters) for right eyes and -1.12 ± 2.02 diopters (median: -0.50 diopter; range: -13.00 to +7.13 diopters) for left eyes. The prevalence of myopia defined as refractive error ≤-0.50D, ≤-1.00D, and ≤-6.00D in the more myopic eye was 58.4 ± 1.3%, 48.0 ± 1.3%, and 3.0 ± 0.4%, respectively. Compared with the participants with available EDI OCT images, the children without EDI OCT images were significantly older (11.8 ± 3.5 versus 12.8 ± 2.8 years; *P* = 0.004) and had a higher proportion of non-Han Chinese ethnicity (23.0% versus 67.6%; *P*<0.001). Both groups did not differ significantly in gender (51.0% versus 54.0% boys; *P* = 0.57) and in refractive error (-1.20 ± 2.03 diopters versus -1.01 ± 1.95 diopters; *P* = 0.37).

The mean subfoveal choroidal thickness was 282 ± 49 μm and 281 ± 51μm for right and left eyes, resp., with no significant difference between both eyes (*P* = 0.80). Choroidal thickness was thickest at 1000μm temporal to the fovea (286 ± 49μm), followed by the subfoveal region (282 ± 49 μm; *P*<0.001), the region at 2500μm temporal to the fovea (278 ± 49μm; *P*<0.001), the region at 1000μm nasal to the fovea (254 ± 49μm, *P*<0.001) and the region at 2500μm nasal to the fovea (197 ± 50μm; *P*<0.001) (Table 1).

**Table 1:** Choroidal thickness (μm) at different retinal locations in the Gobi Desert Children Eye Study.

	Subfoveal Choroidal Thickness (μm) (Right Eye)	Choroidal Thickness at 1000μm Nasal to the Fovea (Right Eye)	Choroidal Thickness at 2500μm Nasal to the Fovea (Right Eye)	Choroidal Thickness at 1000μm Temporal to the Fovea (Right Eye)	Choroidal Thickness at 2500μm Temporal to the Fovea (Right Eye)	Subfoveal Choroidal Thickness (μm) (Left Eye)	Choroidal Thickness at 1000μm Nasal to the Fovea (Left Eye)	Choroidal Thickness at 2500μm Nasal to the Fovea (Left Eye)	Choroidal Thickness at 1000μm Temporal to the Fovea (Left Eye)	Choroidal Thickness at 2500μm Temporal to the Fovea (Left Eye)
n	1463	1463	1463	1463	1463	1463	1463	1462	1463	1463
Mean	282	254	197	286	278	281	256	202	282	268
Median	284	256	196	287	278	283	257	203	282	269
Standard Deviation	49	49	50	49	49	51	51	50	50	47
Minimal Value	91	87	60	117	111	105	83	61	26	13
Maximum Value	417	407	388	430	431	473	437	392	466	442

**Fig 1:**
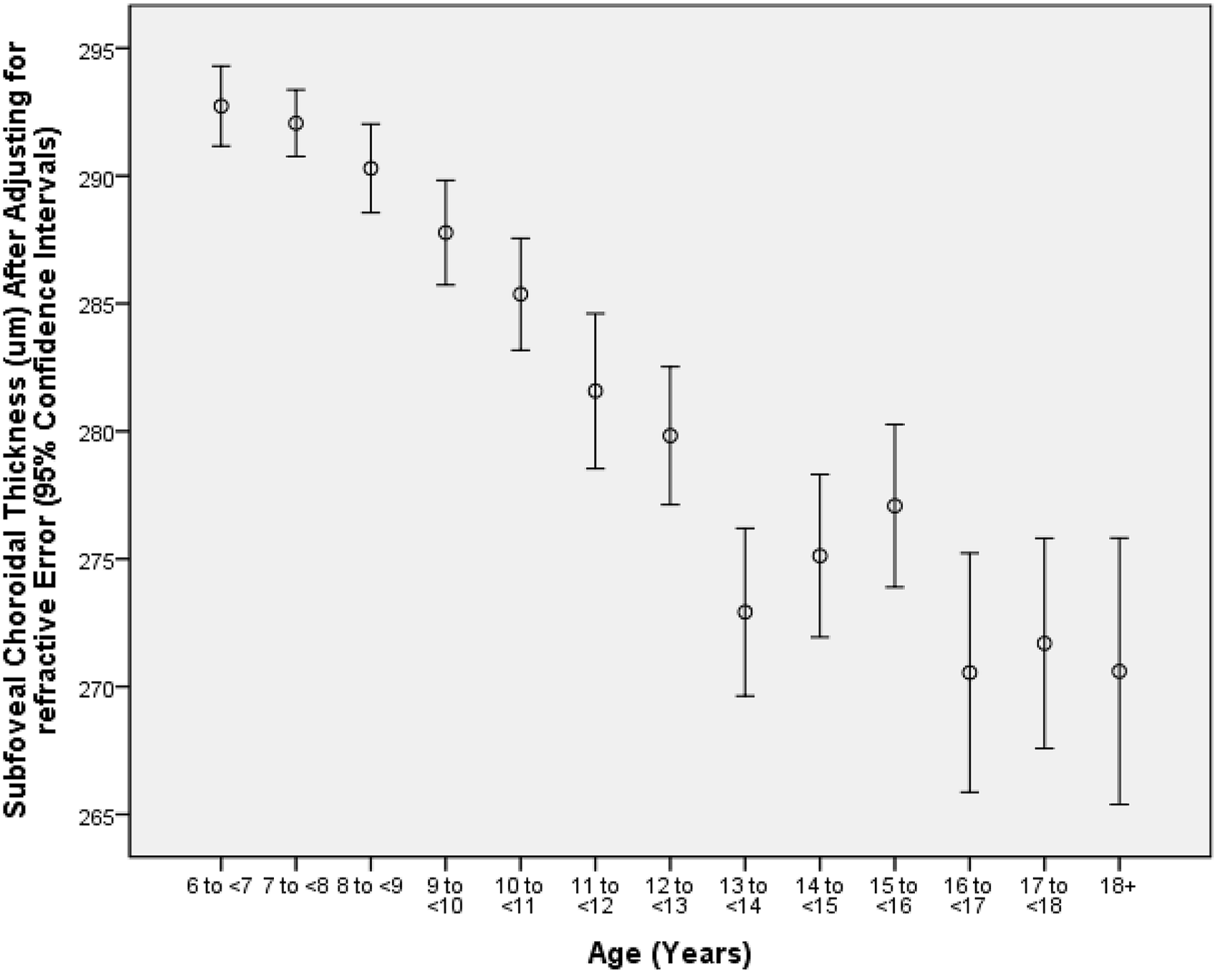
Subfoveal choroidal thickness (After Adjusting for Refractive Error) Stratified by age in the Gobi Desert Children Eye Study.

In the cross-sectional analysis, the mean subfoveal choroidal thickness increased with older age from 288 μm at the age of 7 years to 304 μm at the age of 11 years. At an older age, choroidal thickness decreased with higher age, starting from a choroidal thickness of 298 μm at the age of 12 years to a choroidal thickness of 258 μm at the age of 18+ years (Table 2). The dip of the SFCT curve at the age of 14 was related to the significant increase in myopia in the same age group (Fig 1). The mean refractive error for the age groups of 12, 13, 14 and 15 years was -1.30, -1.52, -2.31 and -2.17 diopters, resp. (Table 2). In univariate analysis, subfoveal choroidal thickness was significantly associated with the systemic parameters of younger age (*P*<0.001), male gender (*P*<0.001), non-Han Chinese ethnicity (*P* = 0.003), lower body height (*P*<0.001), lower body weight (*P*<0.001), lower body mass index (*P*<0.001), lower systolic blood pressure (*P*<0.001), lower diastolic blood pressure (*P*<0.001), lower mean blood pressure (*P*<0.001) and higher pulse rate (*P* = 0.001), and with the ocular parameters of higher hyperopic refractive error (*P*<0.001) and higher corneal refractive power (*P*<0.05) (Table 3).

**Table 2:** Subfoveal choroidal thickness (μm), prevalence of myopia and mean refractive error (spherical equivalent, diopters) in the Gobi Desert Children Eye Study.

Age (Years)	n	Mean	Standard Deviation	Minimum	Maximum	Prevalence of Myopia	Mean Spherical Equivalent
7.00	108	288	38	196	385	.20	.18
8.00	145	290	38	192	386	.23	.05
9.00	140	293	44	202	414	.36	-.20
10.00	148	296	45	205	402	.46	-.51
11.00	112	304	44	172	408	.58	-.81
12.00	140	298	55	166	417	.64	-1.30
13.00	117	280	54	91	407	.70	-1.52
14.00	124	251	51	130	374	.83	-2.31
15.00	100	267	43	142	375	.76	-2.17
16.00	105	275	54	140	399	.78	-1.90
17.00	78	273	47	144	352	.87	-2.58
18+	146	258	46	126	362	.78	-2.24
Total	1463	282	49	91	417	.58	-1.20

**Table 3:** Univariate analysis for associations of subfoveal choroidal thickness in the Gobi Desert Children Eye Study.

Associations	*P*-Value	Standardized Regression Coefficient Beta
Age (Years)	<0.001	-0.24
Gender	0.10	-0.04
Ethnicity	0.003	0.08
Body Height (cm)	<0.001	-0.22
Body Weight (kg)	<0.001	-0.18
Body Mass Index (kg(m^2^)	<0.001	-0.09
Systolic Blood Pressure (mmHg)	<0.001	-0.14
Diastolic Blood Pressure (mmHg)	<0.001	-0.11
Mean Blood Pressure (mmHg)	<0.001	-0.13
Pulse Rate	0.001	0.09
Birth Weight (g)	0.46	0.02
Refractive Error (Diopters)	<0.001	0.34
Best corrected ETDRS VA of right eye	0.80	0.01
Corneal keratometry, Steep Meridian (Diopters)	0.01	0.07
Corneal keratometry, Flat Meridian (Diopters)	0.027	0.06
Intraocular Pressure (mmHg)	0.22	0.03
Time Spent Outdoors (Hours)	0.67	-0.01
Time Spent Indoors (Hours)	0.14	-0.04

The multivariate analysis included subfoveal choroidal thickness as dependent variable and as independent variables all those parameters which were significantly with choroidal thickness in the univariate analysis. Due to collinearity, we first dropped step-by-step body weight (variance inflation factor (VIF): 63.3) and body height (VIF: 6.1). Due to a lack of statistical significance, we then dropped mean blood pressure (*P* = 0.24), systolic blood pressure (*P* = 0.24), pulse rate (*P* = 0.38), mean blood pressure (*P* = 0.53) and body mass index (*P* = 0.06). In the final model, thicker subfoveal choroidal thickness remained to be significantly (regression coefficient r: 0.38) associated with younger age (*P*<0.001; beta: -0.10; B: -1.39; 95%CI: -2.14, -0.64), male gender (*P* = 0.03; beta: -0.05; B: -5.33; 95%CI: -10.1, -0.53), higher hyperopic refractive error (*P*<0.001; beta: 0.31; B: 7.61; 95%CI: 6.29, 8.93), higher corneal refractive power (*P*<0.001; beta: 0.12; B: 3.68; 95%CI: 2.12, 5.24), and non-Han Chinese ethnicity (*P* = 0.03; beta: 0.05; B: 6.16; 95%CI: 0.50, 11.8) (Table 4). If the total time spent indoors (*P* = 0.80) and the total spent outdoors (*P* = 0.16) were added to the multivariate analysis, both parameters were not significantly associated with subfoveal choroidal thickness.

**Table 4:** Multivariate analysis for associations of subfoveal choroidal thickness in the Gobi Desert Children Eye Study.

Parameters	*P*-Value	Standardized Regression Coefficient Beta	Non-Standardized Regression Coefficient B	95% Confidence intervals of B	Variance Inflation Factor
Age (Years)	<0.001	-0.10	-1.39	-2.14, -0.64	1.25
Gender (Men / Women)	0.03	-0.05	-5.33	-10.1, -0.53	1.04
Refractive Error (Diopters)	<0.001	0.31	7.61	6.29, 8.93	1.25
Corneal Refractive Power (Diopters)	<0.001	0.12	3.68	2.12, 5.24	1.06
Ethnicity (Han-Chinese / Others)	0.03	0.05	6.16	0.50, 11.8	1.02

As thicker subfoveal choroidal thickness, thicker choroidal thickness at 2500 μm nasal to the fovea increased (r: 0.36) with younger age (*P* = 0.003; beta: -0.08; B: -1.16; 95%CI: -1.93, -0.38), higher hyperopic refractive error (*P*<0.001; beta: 0.30; B: 7.50; 95%CI: 6.15, 8.86), higher corneal refractive power (*P*<0.001; beta: 0.11; B: 3.70; 95%CI: 2.10, 5.30), and non-Han Chinese ethnicity (*P* = 0.009; beta: 0.07; B: 7.74; 95%CI: 1.92, 13.6), while it was not significantly associated with gender (*P* = 0.14; beta: -0.04; B: -3.70; 95%CI: -8.64, 1.25). If the total time spent indoors (*P* = 0.50) and the total spent outdoors (*P* = 0.91) were added to the multivariate analysis, both parameters were not significantly associated with subfoveal choroidal thickness. Thicker choroidal thickness at 2500 μm temporal to the fovea increased (r: 0.24) with male gender (*P* = 0.001; beta: -0.09; B: -8.60; 95%CI: -13.5, -3.66), higher hyperopic refractive error (*P*<0.001; beta: 0.19; B: 4.65; 95%CI: 3.29, 6.00) and higher corneal refractive power (*P* = 0.004; beta: 0.08; B: 2.35; 95%CI: 0.75, 3.95), while it was not significantly associated with ethnicity (*P* = 0.06; beta: 0.05; B: 5.70; 95%CI: -0.12, 11.5) and age (*P* = 0.15; beta: -0.04; B: -0.56; 95%CI: -1.34, 0.21). If the total time spent indoors (*P* = 0.34) and the total time spent outdoors (*P* = 0.46) were added to the multivariate analysis, both parameters were not significantly associated with choroidal thickness at 2500 μm temporal to the fovea.

Mean ratio of choroidal thickness at 1000 μm nasal to the fovea to subfoveal choroidal thickness (0.90 ± 0.06; range: 0.66, 1.23) decreased with older age (*P* = 0.01; beta: -0.07; B: -0.001; 95%CI: -0.002, 0.000). If the total time spent indoors (*P* = 0.60) and the total time spent outdoors (*P* = 0.39) were added to the multivariate analysis, both parameters were not significantly associated with choroidal thickness. In a similar manner, mean ratio of choroidal thickness at 2500 μm nasal to the fovea to subfoveal choroidal thickness (0.70 ± 0.13; range: 0.28, 1.23) decreased with older age (*P* = 0.001; beta: -0.09; B: -0.003; 95%CI: -0.005, 0.001). If the total time spent indoors (*P* = 0.20) and the total time spent outdoors (*P* = 0.18) were added to the multivariate analysis, both parameters were not significantly associated with choroidal thickness.

**Fig 2:**
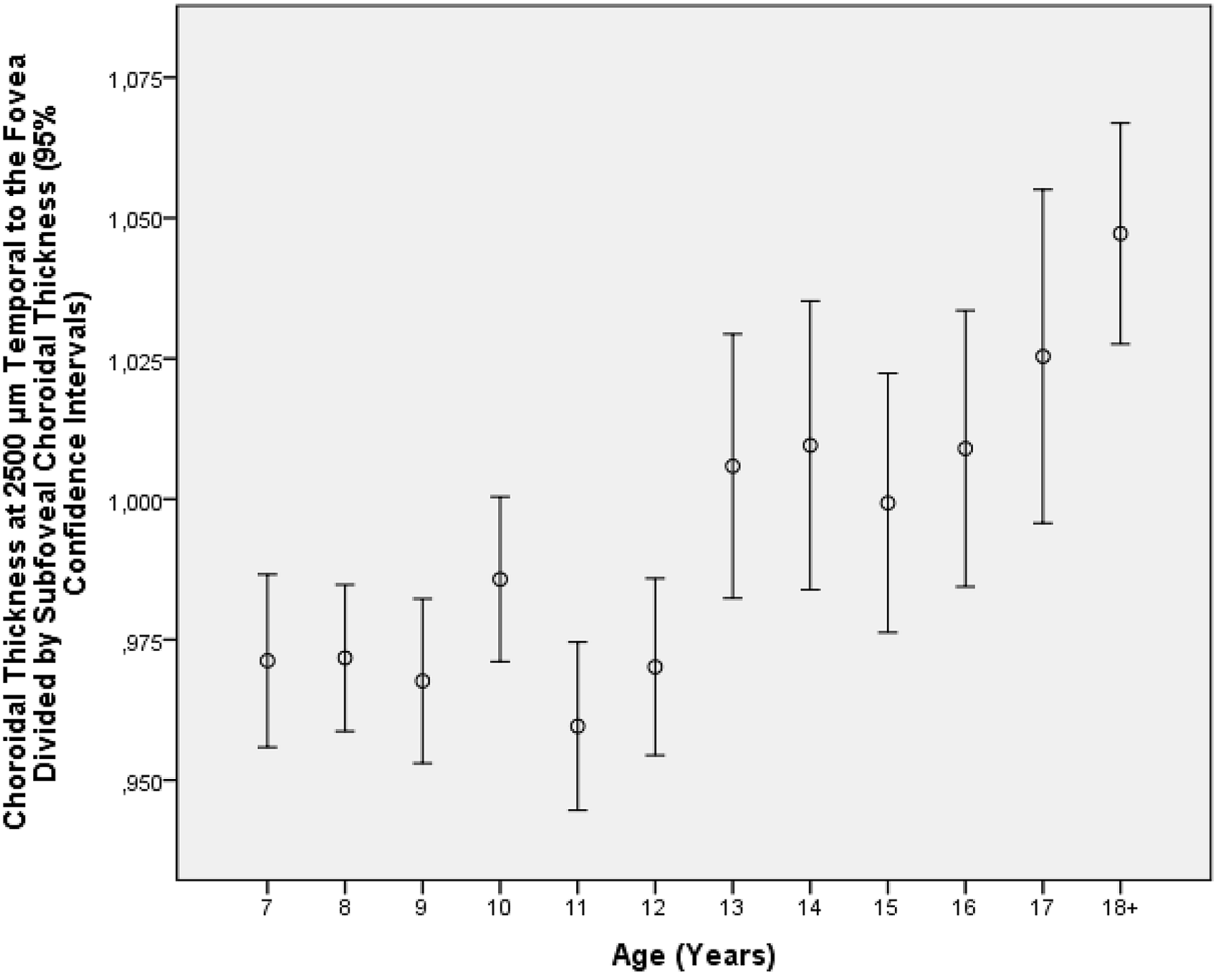
Graph showing the association between age and the ratio of choroidal thickness at 2500 μm temporal to the fovea and subfoveal choroidal thickness in the Gobi Desert Children Eye Study.

The mean ratio of choroidal thickness at 1000 μm temporal to the fovea to subfoveal choroidal thickness (1.02 ± 0.06; range: 0.56, 1.37) increased with older age (*P*<0.001; beta: 0.17; B: 0.003; 95%CI: 0.002, 0.003). If the total time spent indoors (*P* = 0.43) and the total time spent outdoors (*P* = 0.22) were added to the multivariate analysis, both parameters were not significantly associated with choroidal thickness. The mean ratio of choroidal thickness at 2500 μm temporal to the fovea to subfoveal choroidal thickness (0.99 ± 0.11; range: 0.54, 1.84) increased with older age (*P*<0.001; beta: 0.21; B: 0.007; 95%CI: 0.005, 0.008) (Fig 2). If the total time spent indoors (*P* = 0.20) and the total time spent outdoors (*P* = 0.18) were added to the multivariate analysis, both parameters were not significantly associated with choroidal thickness.

## Discussion

In the children of our cross-sectional school-based study, mean choroidal thickness was thickest at 1000μm temporal to the fovea (286 ± 49μm), followed by the subfoveal region (282 ± 49 μm; *P*<0.001), the region at 2500μm temporal to the fovea (278 ± 49μm), the region at 1000μm nasal to the fovea (254 ± 49μm, *P*<0.001) and the region at 2500μm nasal to the fovea (197 ± 50μm; *P*<0.001). In cross-sectional analysis, mean subfoveal choroidal thickness increased with age from 288 μm at 7 years of age to 304 μm at 11 years of age, and then decreased to 258 μm at an age of 18 years. Thicker subfoveal choroidal thickness was associated with higher hyperopic refractive error (*P*<0.001), younger age (*P*<0.001), higher corneal refractive power (*P*<0.001), male gender (*P* = 0.03) and non-Han Chinese ethnicity (*P* = 0.03). Mean ratio of choroidal thickness at locations nasal to the fovea to subfoveal choroidal thickness decreased with older age, while the mean ratio of choroidal thickness at locations temporal to the fovea to subfoveal choroidal thickness increased with older age. Time spent outdoors or time spent indoors was not significantly associated with any choroidal thickness parameter in multivariate analysis.

The mean thickness of the subfoveal choroid in our study population differed from the values reported in some previous studies. In the Copenhagen Child Cohort 2000 Eye Study, mean subfoveal choroidal thickness was 369 ± 81 μm in girls and 348 ± 72 μm in boys, which was higher than the mean value of 282 μm found in our study [30]. Potential reasons for the discrepancy may have been differences in age, refractive errors and ethnic background of the study participants. The Copenhagen Study included children aged 11–12 years and with a mean refractive error of +0.1 diopter, while the age of our study population varied from 7 to 21 years and the mean refractive error was -1.2 diopters. Using Swept source OCT, Nagasawa and colleagues examined 100 healthy Japanese children aged 3–15 years old and reported on a mean choroidal thickness of 260 ± 57 μm [29]. The measurement of subfoveal choroidal thickness reported from our study was almost identical to the value found in the Shandong Children Eye Study on 972 children with a mean age of 11.3 ± 3.3 years (range: 6–18 years), a mean axial length of 24.1 ± 1.6 mm (range: 16.6–28.8 mm) and a mean subfoveal choroidal thickness of 283 ± 67μm (range: 113–507μm) [31].

As in our study, the Shandong Children Eye Study revealed that the choroidal thickness was thicker (*P*<0.001) at 500 μm temporal to the foveola (290 ± 67μm) than in the subfoveal region (283 ± 67μm) and that it was thinnest (*P*<0.001) at 500μm nasal of the foveola (268 ± 67μm). In the investigation performed by Read and colleagues, 4- to 6-year-old children showed the thickest choroid (322 ± 60 μm) 1.5 mm superior to the foveal center. For the 7- to 9-year-olds the mean thickest choroid (344 ± 63 μm) was located in a superior-temporal location 0.8 mm from the foveal center. The thickest choroid of the 10- to 12-year-olds (350 ± 58 μm) was located along 0.9 mm temporal to the foveal center [33]. Sanchez-Cano and associates reported for young adults, that choroidal thickness was thickest in the region 1.5 mm superior to the foveola, followed by the temporal region and the subfoveal region [41]. In another study by Read on children, choroidal thickness was significantly the thickest (346μm) in the superior region and superior-temporal (341μm) location at a distance of 1 to 3 mm from the foveal center, and it was thinnest in the nasal region and inferior-nasal (306μm) area [35]. These findings were different from the observations made in adults, in whom the choroid was usually thickest in the subfoveal region, followed by the temporal region and superior region, and in whom choroidal thickness was thinnest in the nasal perifoveal region [7,8,42]. If the regional distribution of choroidal thickness is compared between children and adults, one may infer, as discussed recently, that the fovea of the retina in spatial relationship to the choroid may move into the temporal direction or that choroidal thickness locally adapts to the eventual location of the fovea in adults [31]. The increase in the ratio of temporal choroidal thickness to subfoveal choroidal thickness with older age up to an age of at least 18 years as shown in our study population may suggest that the re-arrangement of the choroid in terms of moving the location of the thickest choroidal thickness to the subfoveal region may occur after the age of 18 years (Fig 2). The ratio of nasal choroidal thickness to subfoveal choroidal thickness decreased in our young study population. It may be of interest for the discussion on the development of parapapillary alpha, beta and gamma zones, for which a thinning of the choroid has been described [43,44].

The findings of our study agree with the observations made in previous investigations that choroidal thickness decreased with more myopic refractive error or with a longer axial length as surrogate for myopia, with female gender and with older age [7–10,30,31,45–48]. The potential difference between adults and children may be that in adults, choroidal thickness decreased more or less linearly with older age, while in the children of our study population choroidal thickness increased up to an age of 11 years and then started to decrease (Table 2). These results confirmed the findings obtained in previous smaller studies. Read and associates reported that the choroidal thickness increased with older age in a group of 194 children with an age of 4–12 years and in another group of 80 children aged 10–15 years [33,35]. Bidaut-Garnier *et al*. examined 174 children with an age of 3.5 to 15 years and also found an increase in choroidal thickness with older age [36]. In a longitudinal study on 101 children aged 10 to 15 years observed over an 18-month period, Read and colleagues found a significant (*P*<0.001) mean increase of 13 ± 22 μm in subfoveal choroidal thickness in hyperopic eyes and in myopic eyes, in addition to an association between thinner choroidal thickness and axial elongation [46]. In contrast, Nagasawa and colleagues reported that choroidal thickness decreased with age in their group of 100 children with an age of 3 to 15 years [29]. Chhablani and colleagues investigated 136 children with an age of 5–18 years and reported that the choroidal thickness decreased with age [49]. Lee and coworkers reported subfoveal choroid is prone to thinning with increasing age in a group of 40 children with an age of 4–17 years [50]. In our study with a larger sample size, a larger age range and in particular, with a population-based recruitment of the study participants, the mean subfoveal choroidal thickness increased with older age from 288 μm at the age of 7 years to 304 μm at the age of 11 years, and then started to decrease with further ageing to 258 μm at an age of 18 years. These age-related changes in choroidal thickness in association with age-related changes in choroidal thickness may potentially play a role in the yet unclear process of emmetropization and myopization [1,2]. Since intraocular pressure may also influence choroidal thickness and since intraocular pressure also changes with older age in children, future studies may address the inter-relationship between these parameters of axial (optical) length, age, refractive error, intraocular pressure and subfoveal macular choroidal thickness [28].

As in adults, choroidal thickness in the children of our study population as well as in the populations of other children studies decreased with more myopic refractive error or with longer axial length. The Copenhagen Child Cohort 2000 Eye Study reported that a thinner choroidal thickness was associated with more myopic refractive error or shorter axial length [30]. Measuring choroidal thickness and axial length in 160 children, Zengin and associates reported that choroidal thickness was negatively associated with axial length [37]. Similar findings were reported by Herrera *et al*. and by Mapelli and coworkers [47,48]. In our children study population, subfoveal choroidal thickness decreased by 9.5 μm (95%CI: 7.8, 10.3) for each year increase in myopic refractive error in univariate analysis, and by 7.6 μm (95%CI: 6.3, 8.9) for each year increase in myopic refractive error in multivariate analysis (Table 4). In the Beijing Eye Study on adult individuals, subfoveal choroidal thickness decreased by 15.7 μm (95%CI: 13.9, 17.5) for every increase in myopic refractive error of 1 diopter beyond a refractive error of -1 diopter [9].

The associations between male gender and thicker choroidal thickness as found in our study has also been reported for adults and in children. In the Beijing Eye Study and the Singapore Malay Eye Study, subfoveal choroidal thickness was thicker in men than in women [8,9]. In the Shandong Children eye Study, thicker choroidal thickness was associated with male gender, while in the study by Bidaut-Garnier and colleagues on a smaller group of children, choroidal thickness was independent of gender [31,36].

Potential limitations of our study should be mentioned. First, since the study was performed in an oasis city which was definitely not representative for China, it did not supply normative data for the Chinese population. Second, although the Gobi Desert Children Eye Study had a reasonable response rate of 81.9%, the non-participants might have induced a selection bias. Third, only a horizontal OCT scan was performed, so that the topography of the choroidal thickness superior and inferior to the fovea was not assessed. Fourth, the participants of our study underwent the OCT examinations at various times of the day; the effect of circadian (diurnal) rhythm on choroidal thickness was not controlled in the study [51]. However, since these examinations were performed in a randomized manner with respect to what time they were performed, it might have been unlikely that the examination time introduced a bias. Fifth, the interview of parents on children’s indoor/outdoor activity may be a rough and subjective estimation and measuring the refractive power of the glasses worn by parents may not be the most accurate or reliable approach to assess the parents’ refractive error. However, this weakness in the study design may not have markedly affected the SFCT measurements or their interpretation. Sixth, previous investigations have suggested that myopic defocus may cause choroidal thickening, at least in chicken [1]. In our study population, the prevalence of myopia increased with older age, and some children did not wear their best correcting glasses. It may have led to the situation, that a myopic defocus was present in the older children of our study population and that this myopic defocus could potentially have produced a choroidal thickening. Seventh, axial elongation in myopia is associated with an increase in the vertical and horizontal globe diameters (about 0.20 mm per 1 mm axial elongation) [52]. It leads to an increase in the inner scleral surface area with increasing axial length, and secondary, due to geometrical reasons, to a thinning of the choroid. The thinning of the choroid with longer axial length may therefore, at least partially, be due to geometrical reasons.

In conclusion, in our cross-sectional study subfoveal choroidal thickness in children, in contrast to subfoveal choroidal thickness in adults and despite elongating axial length, showed a positive correlation with older age up to 11 years of age and then showed a negative correlation with further ageing until an age of 18 years. It suggests a positive relationship of choroidal volume with age up to the age of 11 years. In children, the choroid was thickest at 1000μm temporal to the fovea, followed by the subfoveal region, and this difference significantly increased with older age. In contrast, choroidal thickness nasal to the fovea in relationship to SFCT decreased with older age. Choroidal thickness in children was independent of lifestyle-associated parameters such as time spent indoors or outdoors.

## References

[1] Nickla DL, Wallman J. The multifunctional choroid. Prog Retin Eye Res. 2010;29:144–168. doi: 10.1016/j.preteyeres.2009.12.002 20044062 10.1016/j.preteyeres.2009.12.002 PMC2913695

[2] Summers JA. The choroid as a sclera growth regulator. Exp Eye Res. 2013;114:120–127. doi: 10.1016/j.exer.2013.03.008 23528534 10.1016/j.exer.2013.03.008 PMC3724760

[3] He L, Frost MR, Siegwart JT Jr, Norton TT. Gene expression signatures in tree shrew choroid during lens-induced myopia and recovery. Exp Eye Res. 2014 6;123:56–71. doi: 10.1016/j.exer.2014.04.005 24742494 10.1016/j.exer.2014.04.005 PMC4155741

[4] He L, Frost MR, Siegwart JT Jr, Norton TT. Gene expression signatures in tree shrew choroid in response to three myopiagenic conditions. Vision Res. 2014 9;102:52–63. doi: 10.1016/j.visres.2014.07.005 25072854 10.1016/j.visres.2014.07.005 PMC4160062

[5] Spaide RF, Koizumi H, Pozzoni MC. Enhanced depth imaging spectral-domain optical coherence tomography. Am J Ophthalmol. 2008;146:496–500. doi: 10.1016/j.ajo.2008.05.032 18639219 10.1016/j.ajo.2008.05.032

[6] Spaide RF. Age-related choroidal atrophy. Am J Ophthalmol. 2009;147:801–810. doi: 10.1016/j.ajo.2008.12.010 19232561 10.1016/j.ajo.2008.12.010

[7] Ikuno Y, Kawaguchi K, Nouchi T, Yasuno Y. Choroidal thickness in healthy Japanese subjects. Invest Ophthalmol Vis Sci. 2010;51:2173–2176. doi: 10.1167/iovs.09-4383 19892874 10.1167/iovs.09-4383

[8] Ding X, Li J, Zeng J, Ma W, Liu R, Li T, et al Choroidal thickness in healthy Chinese subjects. Invest Ophthalmol Vis Sci. 2011;52:9555–9560. doi: 10.1167/iovs.11-8076 22058342 10.1167/iovs.11-8076

[9] Wei WB, Xu L, Jonas JB, Shao L, Du KF, Wang S, et al Subfoveal choroidal thickness: the Beijing Eye Study. Ophthalmology. 2013;120:175–180. doi: 10.1016/j.ophtha.2012.07.048 23009895 10.1016/j.ophtha.2012.07.048

[10] Gupta P, Jing T, Marziliano P, Cheung CY, Baskaran M, Lamoureux EL, et al Distribution and determinants of choroidal thickness and volume using automated segmentation software in a population-based study. Am J Ophthalmol. 2015;159:293–301. doi: 10.1016/j.ajo.2014.10.034 25447120 10.1016/j.ajo.2014.10.034

[11] Shen L, You QS, Xu X, Gao F, Zhang Z, Li B, et al Scleral and choroidal thickness in secondary high axial myopia. Retina. 2016 1 5. [Epub ahead of print] 10.1097/IAE.0000000000000947 26735565

[12] Shao L, Xu L, Wei WB, Chen CX, Du KF, Li XP, et al Visual acuity and subfoveal choroidal thickness. The Beijing Eye Study. Am J Ophthalmol. 2014;158:702–709. doi: 10.1016/j.ajo.2014.05.023 24878308 10.1016/j.ajo.2014.05.023

[13] Kim YT, Kang SW, Bai KH. Choroidal thickness in both eyes of patients with unilaterally active central serous chorioretinopathy. Eye. 2011;25:1635–1640. doi: 10.1038/eye.2011.258 22020172 10.1038/eye.2011.258 PMC3234484

[14] Maruko I, Iida T, Sugano Y, et al Subfoveal choroidal thickness in fellow eyes of patients with central serous chorioretinopathy. Retina. 2011;31:1603–1608. doi: 10.1097/IAE.0b013e31820f4b39 21487334 10.1097/IAE.0b013e31820f4b39

[15] Koizumi H, Yamagishi T, Yamazaki T, et al Subfoveal choroidal thickness in typical age-related macular degeneration and polypoidal choroidal vasculopathy. Graefes Arch Clin Exp Ophthalmol. 2011;249:1123–1128. doi: 10.1007/s00417-011-1620-1 21274555 10.1007/s00417-011-1620-1

[16] Chung SE, Kang SW, Lee JH, Kim YT. Choroidal thickness in polypoidal choroidal vasculopathy and exudative age-related macular degeneration. Ophthalmology. 2011;118:840–845. doi: 10.1016/j.ophtha.2010.09.012 21211846 10.1016/j.ophtha.2010.09.012

[17] Kim SW, Oh J, Kwon SS, et al Comparison of choroidal thickness among patients with healthy eyes, early age-related maculopathy, neovascular age-related macular degeneration, central serous chorioretinopathy, and polypoidal choroidal vasculopathy. Retina. 2011;31:1904–1911. doi: 10.1097/IAE.0b013e31821801c5 21878855 10.1097/IAE.0b013e31821801c5

[18] Jirarattanasopa P, Ooto S, Nakata I, et al Choroidal thickness, vascular hyperpermeability, and complement factor H in age-related macular degeneration and polypoidal choroidal vasculopathy. Invest Ophthalmol Vis Sci. 2012;53:3663–3672. doi: 10.1167/iovs.12-9619 22570352 10.1167/iovs.12-9619

[19] Cao XS, Peng XY, You QS, Zhang YP, Jonas JB. Choroidal thickness in idiopathic subfoveal choroidal neovascularization. Ophthalmologica. 2014;231:221–225. doi: 10.1159/000357114 24603209 10.1159/000357114

[20] Cao XS, Peng XY, You QS, Zhang YP, Jonas JB. Subfoveal choroidal thickness change after intravitreal ranibizumab for idiopathic choroidal neovascularization. Retina. 2014;34:1554–1559. doi: 10.1097/IAE.0000000000000122 24667570 10.1097/IAE.0000000000000122

[21] Schuster AK, Steinmetz P, Forster TM, Schlichtenbrede FC, Harder BC, Jonas JB. Choroidal thickness in non-arteritic anterior ischemic optic neuropathy. Am J Ophthalmol. 2014;158:1342–1347. doi: 10.1016/j.ajo.2014.09.008 25217855 10.1016/j.ajo.2014.09.008

[22] Jonas JB, Forster TM, Steinmetz P, Schlichtenbrede FC, Harder BC. Choroidal thickness in age-related macular degeneration. Retina. 2014 6;34(6):1149–55. doi: 10.1097/IAE.0000000000000035 24220257 10.1097/IAE.0000000000000035

[23] Wang YX, Xu L, Shao L, Zhang YQ, Yang H, Wang JD, et al Subfoveal choroidal thickness in glaucoma. The Beijing Eye Study 2011. PLoS One. 2014;9:e107321 doi: 10.1371/journal.pone.0107321 25210857 10.1371/journal.pone.0107321 PMC4161421

[24] Jonas JB, Steinmetz P, Forster T, Schlichtenbrede FC, Harder B. Choroidal thickness in open-angle glaucoma. J Glaucoma. 2015;24:619–623. doi: 10.1097/IJG.0000000000000063 25415643 10.1097/IJG.0000000000000063

[25] Xu J, Xu L, Du KF, Shao L, Chen CX, Zhou JQ, et al Subfoveal choroidal thickness in diabetes and diabetic retinopathy. The Beijing Eye Study 2011. Ophthalmology. 2013;120:2023–2028. doi: 10.1016/j.ophtha.2013.03.009 23697958 10.1016/j.ophtha.2013.03.009

[26] Jonas JB, Wang N, Wang YX, You QS, Xie XB, Yang D, et al Subfoveal choroidal thickness and cerebrospinal fluid pressure. The Beijing Eye Study 2011. Invest Ophthalmol Vis Sci. 2014;55:1292–1298. doi: 10.1167/iovs.13-13351 24474274 10.1167/iovs.13-13351

[27] Jonas JB, Wang YX, Wei WB, Zhu LP, Shao L, Xu L. Cognitive function and subfoveal choroidal thickness. The Beijing Eye Study. Ophthalmology. 2016;123:220–222. doi: 10.1016/j.ophtha.2015.06.020 26189191 10.1016/j.ophtha.2015.06.020

[28] Wang YX, Jiang R, Ren XL, Chen JD, Shi HL, Xu L, et al Intraocular pressure elevation and choroidal thinning. Br J Ophthalmol. 2016; in Print 10.1136/bjophthalmol-2015-308062 27016503

[29] Nagasawa T, Mitamura Y, Katome T, Shinomiya K, Naito T, Nagasato D, et al Macular choroidal thickness and volume in healthy pediatric individuals measured by swept-source optical coherence tomography. Invest Ophthalmol Vis Sci. 2013;54:7068–7074. doi: 10.1167/iovs.13-12350 24106114 10.1167/iovs.13-12350

[30] Li XQ, Jeppesen P, Larsen M, Munch IC. Subfoveal choroidal thickness in 1323 children aged 11 to 12 years and association with puberty: the Copenhagen Child Cohort 2000 Eye Study. Invest Ophthalmol Vis Sci. 2014;55:550–555. doi: 10.1167/iovs.13-13476 24398094 10.1167/iovs.13-13476

[31] Zhang JM, Wu JF, Chen JH, Wang L, Lu TL, Sun W, et al Macular choroidal thickness in children: The Shandong Children Eye Study. Invest Ophthalmol Vis Sci. 2015;56:7646–7652. doi: 10.1167/iovs.15-17137 26624496 10.1167/iovs.15-17137

[32] Ruiz-Moreno JM, Flores-Moreno I, Lugo F, Ruiz-Medrano J, Montero JA, Akiba M. Macular choroidal thickness in normal pediatric population measured by swept-source optical coherence tomography. Invest Ophthalmol Vis Sci. 2013;54:353–359. doi: 10.1167/iovs.12-10863 23249703 10.1167/iovs.12-10863

[33] Read SA, Collins MJ, Vincent SJ, Alonso-Caneiro D. Choroidal thickness in childhood. Invest Ophthalmol Vis Sci. 2013;54:3586–3593. doi: 10.1167/iovs.13-11732 23652485 10.1167/iovs.13-11732

[34] Park KA, Oh SY. Choroidal thickness in healthy children. Retina. 2013;33:1971–1976. doi: 10.1097/IAE.0b013e3182923477 23644561 10.1097/IAE.0b013e3182923477

[35] Read SA, Collins MJ, Vincent SJ, Alonso-Caneiro D. Choroidal thickness in myopic and nonmyopic children assessed with enhanced depth imaging optical coherence tomography. Invest Ophthalmol Vis Sci. 2013;54:7578–7586. doi: 10.1167/iovs.13-12772 24176903 10.1167/iovs.13-12772

[36] Bidaut-Garnier M, Schwartz C, Puyraveau M, Montard M, Delbosc B, Saleh M. Choroidal thickness measurement in children using optical coherence tomography. Retina. 2014;34:768–774. doi: 10.1097/IAE.0b013e3182a487a4 24013259 10.1097/IAE.0b013e3182a487a4

[37] Zengin MO, Karahan E, Yilmaz S, Cinar E, Tuncer I, Kucukerdonmez C. Association of choroidal thickness with eye growth: a cross-sectional study of individuals between 4 and 23 years. Eye (Lond). 2014;28:1482–1487. 25277304 10.1038/eye.2014.227 PMC4268467

[38] He M, Xiang F, Zeng Y, Mai J, Chen Q, Zhang J, et al Effect of time spent outdoors at school on the development of myopia among children in China: A randomized clinical trial. JAMA. 2015;314:1142–1148. doi: 10.1001/jama.2015.10803 26372583 10.1001/jama.2015.10803

[39] Yang DY, Guo K, Wang Y, Guo YY, Yang XR, Jing XX, et al Intraocular pressure and associations in children. The Gobi Desert Children Eye Study. PLoS One. 2014;9:e109355 doi: 10.1371/journal.pone.0109355 25295855 10.1371/journal.pone.0109355 PMC4190171

[40] Guo K, Yang da Y, Wang Y, Yang XR, Jing XX, Guo YY, et al Prevalence of myopia in schoolchildren in Ejina: the Gobi Desert Children Eye Study. Invest Ophthalmol Vis Sci. 2015;56:1769–1774. doi: 10.1167/iovs.14-15737 25626973 10.1167/iovs.14-15737

[41] Sanchez-Cano A, Orduna E, Segura F, Lopez C, Cuenca N, Abecia E, et al Choroidal thickness and volume in healthy young white adults and the relationships between them and axial length, ammetropy and sex. Am J Ophthalmol. 2014;158:574–583. doi: 10.1016/j.ajo.2014.05.035 24907431 10.1016/j.ajo.2014.05.035

[42] Manjunath V, Taha M, Fujimoto JG, Duker JS. Choroidal thickness in normal eyes measured using Cirrus HD optical coherence tomography. Am J Ophthalmol. 2010;150:325–329. doi: 10.1016/j.ajo.2010.04.018 20591395 10.1016/j.ajo.2010.04.018 PMC2926223

[43] Jonas JB, Jonas SB, Jonas RA, Holbach L, Dai Y, Sun X, et al Parapapillary atrophy: Histological gamma zone and delta zone. PLoS One. 2012;7:e47237 doi: 10.1371/journal.pone.0047237 23094040 10.1371/journal.pone.0047237 PMC3475708

[44] Sullivan-Mee M, Patel NB, Pensyl D, Qualls C. Relationship between juxtapapillary choroidal volume and beta-zone parapapillary atrophy in eyes with and without primary open-angle glaucoma. Am J Ophthalmol. 2015;160:637–647.e1. doi: 10.1016/j.ajo.2015.06.024 26144700 10.1016/j.ajo.2015.06.024 PMC4569512

[45] Barteselli G, Chhablani J, El-Emam S, Wang H, Chuang J, Kozak I, et al Choroidal volume variations with age, axial length, and sex in healthy subjects: a three-dimensional analysis. Ophthalmology. 2012;119:2572–2578. doi: 10.1016/j.ophtha.2012.06.065 22921388 10.1016/j.ophtha.2012.06.065 PMC3514583

[46] Read SA, Alonso-Caneiro D, Vincent SJ, Collins MJ. Longitudinal changes in choroidal thickness and eye growth in childhood. Invest Ophthalmol Vis Sci. 2015;56:3103–3112. doi: 10.1167/iovs.15-16446 26024094 10.1167/iovs.15-16446

[47] Herrera L, Perez-Navarro I, Sanchez-Cano A, Perez-Garcia D, Remon L, Almenara C, et al Choroidal thickness and volume in a healthy pediatric population and its relationship with age, axial length, ametropia and sex. Retina. 2015 6 3. [Epub ahead of print] 10.1097/IAE.0000000000000636 26049622

[48] Mapelli C, Dell'Arti L, Barteselli G, Osnaghi S, Tabacchi E, Clerici M, et al Choroidal volume variations during childhood. Invest Ophthalmol Vis Sci 2013;54:6841–6845. doi: 10.1167/iovs.13-12761 24065815 10.1167/iovs.13-12761

[49] Chhablani JK, Deshpande R, Sachdeva V, Vidya S, Rao PS, Panigati A, et al Choroidal thickness profile in healthy Indian children. Indian J Ophthalmol. 2015;63:474–477. doi: 10.4103/0301-4738.162577 26265634 10.4103/0301-4738.162577 PMC4550976

[50] Lee JW, Song IS, Lee JH, Shin YU, Lim HW, Lee WJ, et al Macular choroidal thickness and volume measured by swept-source optical coherence tomography in healthy Korean children. Korean J Ophthalmol. 2016;30:32–39. doi: 10.3341/kjo.2016.30.1.32 26865801 10.3341/kjo.2016.30.1.32 PMC4742643

[51] Tan CS, Ouyang Y, Ruiz H, Sadda SR. Diurnal variation of choroidal thickness in normal, healthy subjects measured by spectral domain optical coherence tomography. Invest Ophthalmol Vis Sci. 2012;53:261–266. doi: 10.1167/iovs.11-8782 22167095 10.1167/iovs.11-8782

[52] Jonas JB, Ohno-Matsui K, Holbach L, Panda-Jonas S. Association between axial length and horizontal and vertical globe diameters. Graefes Arch Clin Exp Ophthalmol. 2016; In Print 10.1007/s00417-016-3439-2 27473372

